# Contributions of Climate and Soil Properties to Wheat and Maize Yield Based on Long-Term Fertilization Experiments

**DOI:** 10.3390/plants10102002

**Published:** 2021-09-24

**Authors:** Shengbao Wei, Anchun Peng, Xiaomin Huang, Aixing Deng, Changqing Chen, Weijian Zhang

**Affiliations:** 1 Jiangsu Collaborative Innovation Center for Modern Crop Production, Nanjing Agricultural Unversity, Nanjing 210095, China; 2019101014@njau.edu.cn (S.W.); 2020101021@stu.njau.edu.cn (A.P.); 2014201018@njau.edu.cn (X.H.); 2 Institute of Crop Sciences, Chinese Academy of Agricultural Sciences/Key Laboratory of Crop Physiology and Ecology, Ministry of Agriculture, Beijing 100081, China; dengaixing@caas.cn

**Keywords:** climate change, food security, crop production, soil quality, long-term experiment, multiple cropping

## Abstract

Identifying the contributions of climate factors and soil fertility to crop yield is significant for the assessment of climate change impacts on crop production. Three 20-year field experiments were conducted in major Chinese wheat-maize cropping areas. Over the 20-year period, crop yield and soil properties showed significantly dissimilar variation trends under similar climate changes at each experimental site. The correlation between climatic factors and crop yield varied greatly among the fertilization regimes and experimental sites. Across all the fertilization regimes and the experimental sites, the average contribution rates of soil properties to wheat and maize yield were 45.7% and 53.2%, respectively, without considering climate factors, and 40.4% and 36.6%, respectively, when considering climate factors. The contributions of soil properties to wheat and maize yield variation when considering climate factors were significantly lower than those without considering climate factors. Across all experimental sites and all fertilization regimes, the mean contribution rates of climate factors to wheat and maize yield were 29.5% and 33.0%, respectively. The contribution rates of the interaction of climate and soil to wheat and maize yield were 3.7% and −0.9%, respectively. Under balanced fertilization treatments (NPK and NPKM), the change in the contribution rate of soil properties to wheat or maize yield was not obvious, and the average contribution rates of the interaction of climate and soil to wheat and maize yield were positive, at 14.8% and 9.5%, respectively. In contrast, under unbalanced fertilization treatments (CK and N), the contribution rates of soil properties to wheat or maize yield decreased, and the average contribution rates of the interaction of climate and soil were negative, at −7.4% and −11.2%, respectively. The above results indicate that climate and soil synergistically affected crop yields and that, with the optimization of the fertilization regime, positive interactions gradually emerged.

## 1. Introduction

Evidence has shown that climate change is inevitable, and the climate is predicted to change much faster in the coming decades [1]. Climatic factors (e.g., temperature and precipitation) play a key role in controlling crop growth and development; thus, even a moderate change in these climatic factors can significantly affect crop productivity [2]. Furthermore, grains provide approximately 50% of food calories worldwide [3], and wheat and maize are the most important cereal crops [4]. China is the largest grower and consumer of wheat and maize in the world; Chinese wheat and maize production account for 20.9% and 27.9% of global wheat and maize production, respectively [5]. Moreover, major Chinese wheat-maize cropping areas are located in northern areas that use multiple cropping systems, where climate change is anticipated to have a strong effect [1]. Therefore, quantifying the impacts of climate change on Chinese wheat and maize production can provide an important reference that can be used to enhance food security in China and in the world [6,7].

Great efforts have been made to assess climate change impacts on Chinese grain production [7,8]. Based on climate and historical yield data, for example, Chavas et al. [9] reported that future climate change might significantly increase winter wheat productivity in China. Conversely, other studies have shown that future climate change might greatly decrease Chinese wheat yield [10]. Based on the mean yields from 1961 to 1990, climate warming might decrease Chinese winter wheat yield by 6.5–8.6% by the end of 2100 [9]. However, based on the mean yields from 1985 to 2000, a 1.0 °C increase in daily mean temperature might increase Chinese winter wheat yield by 7.8% [11]. Recently, our field warming experiments demonstrated that an increase of less than 1.5 °C in air temperature enhanced Chinese winter wheat yield by more than 10% [12]. Chen et al. [13] found that a 1 °C increase in daily minimum temperature in September might lead to an increment of 283 kg ha^−1^ in maize yield in Northeast China. However, Ju et al. [14] reported that maize yield has been projected to decrease by 0.4–11.9% by 2050. Lobell et al. [10] also showed that with a 1.0 °C increase in temperature, maize yield was reduced by 6% from 1980 to 2008 in China and might continue to decline by 3.8% by 2030 [15]. Apparently, great uncertainties still remain in the assessment of climate change impacts on Chinese wheat and maize production.

Previous studies were mostly based on historical statistical data or long-term experimental data, and they mainly focused on climatic factors, while non-climatic factors were simplified as linear trends [16]. In fact, along with climate change, agronomic practices can also cause great changes in soil fertility (e.g., soil organic carbon, nutrient contents, and pH value), which in turn significantly influences crop productivity and crop responses to climate change. For example, soil acidification caused by chemical nitrogen fertilization has resulted in serious negative impacts on Chinese wheat and maize production during the past decades [17]. Conversely, soil nitrogen additions due to air nitrogen deposition increases might have benefited crop production in China during the same period [18]. Based on the national soil survey, the soil organic matter (SOM) contents of farmland have increased in North China during the last three decades [19], which might have also greatly enhanced wheat and maize productivity. Along with climate changes, all the long-term soil properties changes caused by environmental factors and agronomic practices might either enhance or degrade crop productivity in the long term. In addition, climate change might affect crop growth indirectly by stimulating soil properties changes. In most previous studies, however, the impacts of soil properties changes on crop yield were ignored or simplified. Since climate factors and soil properties change simultaneously, distinguishing the contributions of climate factors and soil properties to crop yield is necessary for the assessment of climate change impacts.

Fertilization is the most common agronomic practice and is a key practice affecting soil properties. Thus, long-term field experiments with different fertilization regimes can provide a good way to separate the impacts of climate and soil properties on crop yields. Moreover, canonical correlation analysis (CCA) is a method often favoured by researchers who are analysing compositional data, which are extremely useful for ecological interpretation [20]. Therefore, based on three 20-year fertilization experiments located in major Chinese wheat-maize cropping areas, a simple correlation analysis and a CCA-based variation partitioning analysis (VPA) were conducted in the present study. The objectives of the analysis were (1) to clarify the relationship between crop yield and climate factors or soil properties, (2) to quantify the impacts of the changes in air temperature, precipitation and the main soil properties index on wheat and maize yield variations under a wheat-maize cropping system, and (3) to introduce complexity to and provide a challenge for climate change research. Our study may provide valuable insights into how global changes and soil properties affect crop yield.

## 2. Results

### 2.1. Variations in Main Climatic Factors

**Figure 1:**
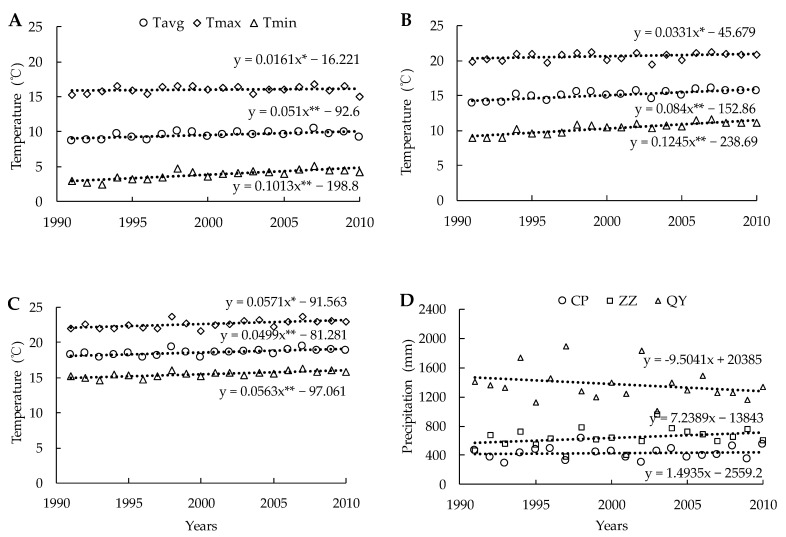
Historical trends of annual average, maximum, minimum temperatures and precipitation in the three experimental sites during 1991–2010. Straight lines are the linear regression lines. (**A**) Changping, (**B**) Zhengzhou, (**C**) Qiyang, (**D**) The historical trends of precipitation. ** Significant at *p* < 0.01; * significant at *p* < 0.05.

The annual mean, minimum and maximum temperatures, and their change trends are presented in Figure 1. Since 1991, the three experimental sites have all experienced obvious warming trends. The increases in mean, maximum and minimum temperatures per 10-year period were 0.51 °C, 0.16 °C, and 1.01 °C for Changping, 0.84 °C, 0.33 °C, and 1.24 °C for Zhengzhou, and 0.50 °C, 0.57 °C, and 0.56 °C for Qiyang, respectively. All the temperatures showed significantly warming trends. The highest increments were found in the daily minimum temperature.

The changes in precipitation are presented in Figure 1D. These trends were not significant, the total precipitation declined by 95.0 mm per 10-year period at the Qiyang, while it increased by 14.9 mm and 72.3 mm since 1991 at the Changping site and Zhengzhou sites, respectively.

### 2.2. Changes in Soil Properties under Different Fertilization Regimes 

There were significant differences in the impacts of long-term fertilization on the topsoil properties (0–20 cm) between the control and the experimental treatments (Table 1). The soil pH values tended to decrease during the experimental period, especially at the Qiyang site, which showed a significant downward trend in pH. The soil organic matter (SOM) content showed a significant rising trend under the NPK and NPKM treatments, while it remained unchanged or even slightly decreased under the other treatments. Similar trends as those of the SOM were found in soil total nitrogen (TN) content under each fertilization regime for all sites. The soil available phosphorus (AP) and potassium (AK) showed increasing trends over the experimental periods under the NPKM treatment, while they tended to decrease under the other treatments, especially the CK and N treatments. 

### 2.3. Variations in Crop Yields 

There were significant differences in the variation and change trends of crop yields for the different crops, treatments, and experimental sites (Table 2). The average yields of both wheat and maize across the experimental years were significantly higher in the NPK and NPKM treatments than in the CK and N treatments for all experimental sites. The average yields of wheat were significantly lower than those of maize for all treatments in the three sites. Except for the NPK treatment at the Qiyang site, wheat and maize yields under the NPK and NPKM treatments showed an increasing trend. The yield of wheat and maize showed significantly decreasing trends under the CK and N treatments. 

### 2.4. Correlation Coefficients between Crop Yields and Climate Factors and Soil Fertility Factors 

The correlation coefficients between the crop yields and the main climatic and soil fertility factors were significantly different among fertilization regimes and experimental locations (Table 3 and Table 4). No significant correlations between the climatic factors and wheat and maize yields were found at the Changping site under any treatment. However, significant negative correlations between the minimum temperature and wheat or maize yield were found at the Zhengzhou and Qiyang sites, except in all NPKM treatments, the CK treatment on wheat in Zhengzhou, and the NPK treatment on maize in Zhengzhou. Moreover, except in the NPKM treatments, most correlation coefficients between crop yield and temperature were negative. There were no significant correlations between precipitation and crop yield.

For the correlations between the soil fertility factors and crop yield, significant correlation coefficients were mostly found for the N treatment, while only a few correlation coefficients were significant under the other treatments. Most of the correlation coefficients between the crop yields and SOM or AP were significantly positive. There were a few significant correlation coefficients between soil TN or AK and crop yield. Among the experimental sites, the highest number of significant correlation coefficients between the soil fertility factors and crop yield were found at the Qiyang site, and there was a strong correlation between pH and crop yield. 

### 2.5. Contributions of Climate and Soil to Crop Yield 

**Figure 2:**
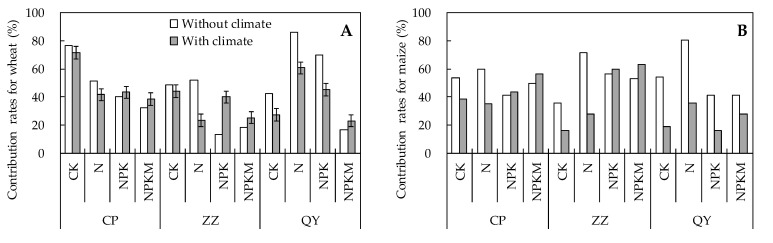
The contribution rates of soil property to the wheat (**A**) and maize (**B**) yields.

According to the CCA-based variation partitioning analysis (VPA), large differences in the contributions of soil properties to crop yield were found between the crops and the fertilization regimes (Figure 2). For all fertilization regimes and the three experimental sites, the average contribution rates of soil properties to wheat yield were 45.7% without considering the climate factors and 40.4% considering the climate factors. For each site across all treatments, without considering the climate factors, the mean contribution rates of soil properties to wheat yield were 50.3%, 33.2%, and 45.8% at Changping, Zhengzhou, and Qiyang, respectively. Considering the climate factors, the mean contribution rates of soil properties to wheat yield were 48.8%, 33.3%, and 39.1% at Changping, Zhengzhou, and Qiyang, respectively. For each treatment across the experimental sites, the corresponding contribution rates of soil properties to wheat yield, with and without considering the climate factors, were 55.9% and 47.7% under CK, 63.2% and 42.0% under N, 41.3% and 42.9% under NPK, and 22.6% and 29.0% under NPKM, respectively. 

For maize yield, the average contribution rates of soil properties were 53.2% without considering the climate factors and 36.6% considering the climate factors for all fertilization regimes and the three experimental sites. For each site across all treatments, without considering the climate factors, the mean contribution rates of soil properties to maize yield were 51.2%, 52.2%, and 54.2% at Changping, Zhengzhou, and Qiyang, respectively. Considering the climate factors, the mean contribution rates of soil properties to maize yield were 43.4%, 41.8%, and 24.7% at Changping, Zhengzhou, and Qiyang, respectively. For each treatment across the experimental sites, the corresponding contribution rates of soil properties to maize yield, with and without considering the climate factors, were 47.7% and 24.7% under CK, 70.5% and 33.0% under N, 46.4% and 39.8% under NPK, and 48.1% and 49.1% under NPKM, respectively.

**Figure 3:**
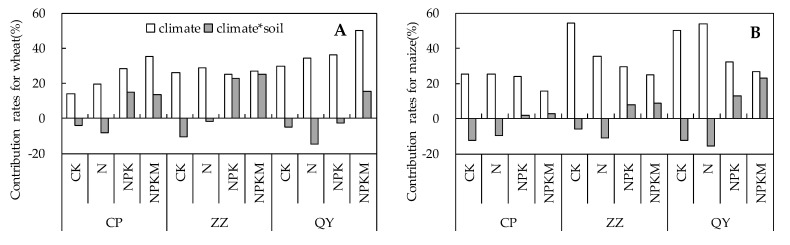
The contribution rates of climate, climate and soil interaction to the wheat (**A**) and maize (**B**) yields.

The average contribution rates of climate were 29.5% and 33.0% to wheat and maize yield, respectively (Figure 3). The average contribution rates of climate and soil interactions were 3.7% and −0.9% to wheat and maize yield, respectively. For each site across all treatments, the mean contribution rates of climate to wheat yield were 24.3%, 26.8%, and 37.5% at Changping, Zhengzhou, and Qiyang, respectively. For each treatment across the experimental sites, the corresponding contribution rates to wheat yield were 23.2% under CK, 27.7% under N, 29.9% under NPK, and 37.3% under NPKM. For maize yield, the mean contribution rates of climate were 22.5%, 35.9%, and 40.6% at Changping, Zhengzhou, and Qiyang, respectively. For each treatment across the experimental sites, the corresponding contribution rates to maize yield were 43.2% under CK, 38.0% under N, 28.6% under NPK, and 22.4% under NPKM. For the interaction of climate and soil, the mean contribution rates to wheat yield were positive at the Changping and Zhengzhou sites, at 4.0% and 8.9%, respectively. However, the interaction of climate and soil had a negative contribution rate of −1.8% at the Qiyang site. For each treatment across the experimental sites, the corresponding contribution rates were −6.7% under CK, −8.2% under N, 11.7% under NPK, and 17.9% under NPKM. For maize yield, the mean contribution rates of the interaction of climate and soil were negative at the Changping and Zhengzhou sites, at −4.3% and −0.1%, respectively, and there was a positive contribution rate of 1.9% at the Qiyang site. For each treatment across the experimental sites, the corresponding contribution rates were −10.3% under CK, −12.1% under N, 7.5% under NPK, and 11.5% under NPKM. 

## 3. Discussion

The response of variation in crop yield to climate varies from region to region, depending on the cropping system, climate, and spatial scale [11]. Both temperature and precipitation are important climatic factors affecting the variability of crop yield, but the main factors affecting crop yield are varied at the different spatial scales [2,21]. The variability of wheat yield is mainly affected by the temperature, in contrast to rainfall at the small scale, and the impact of precipitation on wheat yield becomes weaker with the expansion of the spatial scale [11]. However, crop yield can be significantly affected not only by climatic factors but also by other factors, such as soil fertility and agronomic practices, and by the interactions between factors [22,23]. Thus, under the same climatic change, crop yield can show an increasing, decreasing, or unchanged trend due to the changes in other factors. Since fertilization is a common agronomic practice, field observations based on long-term fertilization experiments can provide a good chance to understand the impacts of climate and soil on crop yield [24]. In our study, the three experimental sites all experienced significant warming trends during the 20-year experimental period (Figure 1). As the climate changed, the soil properties also changed significantly due to the different fertilization regimes (Table 1). Under similar warming trends, interestingly, wheat and maize yields showed dissimilar change trends under the different fertilization regimes (Table 2), indicating that fertilization could affect crop yield in response to climate change. The different trends could be attributed to the impacts of fertilization on soil properties. Other long-term experiments have also demonstrated that differences in crop yield variation trends were mainly attributed to long-term fertilization impacts [25]. Liu et al. [26] also reported that wheat yield trends were variable under different fertilization regimes in the same warming environment. In our long-term fertilization experiment, both wheat and maize yields increased along with the warming trends under appropriate fertilization practices; however, they decreased under inappropriate fertilization practices (Table 2). Consequently, different or even adverse conclusions about the impacts of climate change on crop yield might be drawn for the same site depending on the agronomic practices or soil properties changes at the site. These inconsistencies indicate that more environmental factors need to be considered when assessing the impacts of climate change on crop yield based on historical data.

Crop yields depend on the key factors that limit crop growth, and soil fertility is one of the most important key factors [25]. Thus, the rates of contribution to crop yield of the changes in different environmental factors can vary. In the present study, the contribution rates of soil properties to wheat and maize yields were significantly higher than those of climate, regardless of the experimental site or which fertilization regime was applied (Figure 2 and Figure 3). Moreover, the lowest contribution rates of climate to wheat yield were mostly found under the unbalanced fertilization practices (e.g., the N treatment). This suggests that long-term yield variation trends might depend more on soil properties changes than on climate changes under improper agronomic practices. Inappropriate fertilization practices such as sole N fertilization and unbalanced fertilization can significantly affect soil structure and nutrient availability. A downward trend of the soil pH was observed under the four fertilization regimes at the Qiyang site, which was in agreement with previous findings [27]. The Qiyang site is a typical red loam soil, and fertilization is considered to be one of the reasons for the acidification of red soil, but combining organic fertilizer with chemical fertilizer has an obvious effect on improving soil acidification [27,28]. Moreover, the disturbance of irrigation and vegetation would aggravate the acidification of the red soil [29,30], which may be the cause of the pH decline in the non-fertilized control. The contents of SOM, AP, and AK increased significantly under NPKM treatment at the three experimental sites (Table 1). Meanwhile, except for Zhengzhou site, the highest yield of wheat and maize occurred in the NPKM treatment across the four fertilization regimes (Table 2). The application of organic fertilizer is an important way to improve crop yield and quality [31]. Previous studies have shown that compared to the application of chemical fertilizer alone, chemical fertilizer combined with organic fertilizer can more effective at enhancing crop yield [32,33]. In addition, although long-term chemical fertilization application resulted in higher yield than non-fertilized control at all, the crop yield showed a downward trend over time [27]. SOM and available nutrient increments caused by good agronomic practices can greatly improve soil abiotic and biotic quality [34,35], resulting in high resilience to environmental changes and sustainable increases in crop yield (Table 2). Thus, crop growth might mainly depend on climate factors rather than soil factors under good agronomic practices. On the other hand, incorrect agronomic practices might degrade soil quality and resilience to environmental changes—for instance, the sensitivity of crops to weather may be increased under higher fertilizer applications [36], resulting in more serious limitations from climate factors. Consequently, crop yield might largely depend on changes in the soil conditions. Furthermore, there were significant differences in the contributions of climate and soil properties to crop yield variations between wheat and maize and among the experiment sites (Figure 2 and Figure 3). The differences might also be because of the seasonal and spatial discrepancies in soil and climate conditions (Figure S2 and Table S1). In this study, we found that the yield of wheat and maize at the Qiyang site was the lowest among the three experimental sites. Without considering the climate factors, the mean contribution rates of soil properties to wheat and maize yield were 45.8% and 54.2% in the Qiyang site, respectively (Figure 2). Soil fertility is an important factor limiting crop yields; however, poor soil fertility, nutrient loss caused by leaching, and lower nutrient availability are the common problems in the acidic red loam [37,38], which all negatively impact on crops growth and nutrients uptake, and ultimately cause yield decline. The Changping site is located in the northern boundary of the winter wheat planting area in China. Low temperature and little precipitation are main reasons affecting the growth and yield of winter wheat in the area [39], whereas Zhengzhou is the most favorable site for wheat-maize cropping with high yield of the three experimental sites. Moreover, the climate and soil properties are normally less favourable for wheat growth than for maize growth at the tested sites. Thus, the contributions of climate and soil properties changes to crop yield variation can be different for each specific crop and cropping area.

Crop yields are also significantly affected by the interactions of environmental factors, especially climate and soil factors [26,40]. Climate change might benefit crop growth indirectly by improving soil fertility [41,42]; for example, moderate warming could enhance soil nutrient availability [26,40]. Temperature and nutrient availability are crucial in controlling the pathway and rates at which energy and materials move through ecosystems [43]. In the cold season, rising temperature would promote the formation of easily decomposable organic nitrogen produced under dry conditions, and increase nitrogen mineralization during the next warm season [44]. The plants stimulate the nitrogen cycle and nutrient availability in low-fertility soil by increasing the release of root exudation under experimental warming, so that nutrients can be more readily mineralized and made available to plants [45]. Furthermore, good agronomic practices could mitigate the negative impacts of climate changes on crop production by enhancing soil quality and resilience [36,46]. In the present study, significant differences in the interactions of climate and soil properties changes were found among the fertilization regimes and experimental sites (Figure 3). Moreover, there were great differences in the correlation coefficients of climate change with crop yield variation between the fertilization regimes (Table 3 and Table 4). According to existing studies, some research methods can remove the influence of technological development, such as using a first difference time series [47], nonlinear detrending [10] or multiple regression [48]. However, the interactions of climate and soil properties changes have not been considered in most of the existing methods, which might result in great uncertainties in the assessment of the impacts of climate change on crop production. If the impacts of climate and soil properties changes on crop production trend in the same direction (a positive interaction), the actual impacts of climate change might be over-estimated. However, if the impacts of climate and soil properties changes are in opposite directions (a negative interaction), the real impacts of climate changes might be under-estimated. According to the present analysis, the actual impacts of climate change on crop yield might be under-estimated due to the interactions between climate and soil properties that were negative under inappropriate agronomic practices but might be over-estimated due to the interactions between climate and soil properties that were positive under good agronomic practices (Figure 3).

The present study indicates the strong contributions of soil properties and its interactions with climate to long-term crop yield. The results illustrate the complex effects of different climate scenarios and soil conditions on crop yield and highlight the need for a further understanding of the integrated impacts of soil and climate changes on crop yield. However, this study was mainly based on a 20-year experimental duration at three typical locations in major Chinese wheat-maize cropping areas. For climate change issues, the temporal scale of this study is somewhat limited. More data across a longer time scale are necessary to further assess the mechanisms underlying the integrated impacts of climate and soil properties on crop production. Although the three sites are typical locations in the major Chinese wheat-maize cropping region, the spatial scale of the three sites is also somewhat limited due to the large differences in climate factors among cropping areas. For China and other similar areas, therefore, the results from the present study need to be further investigated across different soil and climate conditions. Moreover, further evidence from field experiments is also necessary to improve the certainty of assessments of future climate change impacts on crop production in China.

## 4. Materials and Methods

### 4.1. Site Description 

The 20-year field experiments were conducted at three sites, Changping (40°13′ N, 116°15′), Zhengzhou (34°47′ N, 112°40′), and Qiyang (26°45′ N, 111°52′), that have been cultivated with an annual wheat-maize cropping system since 1990. The locations of the three long-term experimental sites are shown in Figure S1. The experimental sites belong to the National Soil Fertility and Fertilizer Effects Long-term Monitoring Network (NSFN) of China. The main climatic and soil information is shown in Figure S2 and Table S1 in detail.

### 4.2. Experimental Design 

Four fertilization regimes with three replicates were applied at each site: (1) non-fertilized control (CK), (2) inorganic nitrogen fertilization only (N), (3) balanced fertilization with inorganic nitrogen, phosphorus, and potassium (NPK), and (4) balanced inorganic NPK fertilization with manure application (NPKM). Each replicate plot size was 200 m^2^. At the Changping site from 1990 to 2000, the maize variety used was Zhongdan 120, and the wheat variety used was Zhong 8693; the maize and wheat varieties have been Tangkang 5 and Zhongzuo 9507, respectively, since 2001. At the Zhengzhou site, the wheat varieties used were Yumai 13, Zhengtaiyu 1, Linfen 7203, Zhengzhou 941, Yumai 47, and Zhengzhou 8998 from 1990 to 2000 and Zhengmai 9023 from 2001 to 2010, and the maize variety used was Zhengdan 8. At the Qiyang site, the maize varieties used were Yedan 4 from 1990 to 1994 and Yedan 13 from 1995 to 2010; the wheat varieties used were Xiangmai 12 from 1990 to 1993 and Xiangmai 11 from 1994 to 2010. Wheat was sown in late October and harvested in late May or June of the following year. Maize was sown in early June and harvested in late September. The straw was removed after harvest. The harvested crops were threshed, and the grains were separated to determine the yield in each plot.

Field management practices such as tillage, weeding, irrigation, the application of pesticides, were carried out based on the local agronomic requirements for high yield.

The inorganic fertilizers were urea (N 46%), calcium superphosphate (P_2_O_5_ 12.5%), and potassium sulfate (K_2_O 50%). The organic fertilizer used was swine manure at the Qiyang site and a cow dung and straw mixture at the Zhengzhou and Changping sites. The organic fertilizer used was different every year, and the nitrogen content of the organic fertilizer was analyzed and the rate determined before fertilization. The details of the fertilization regimes conducted for wheat-maize cropping in the experimental sites are shown in Table S2.

### 4.3. Soil Sampling and Analysis

Soil samples were collected after the maize harvest every year. In each plot, six cores were taken to a depth of 20 cm and were mixed as a composite sample. Soil organic matter (SOM), total nitrogen (TN), pH, available phosphorus (AP), and available potassium (AK) in soil samples were determined. Soil organic matter was determined using a wet oxidation method with K_2_Cr_2_O_7_ and concentrated H_2_SO_4_. Soil pH was determined in a soil-to-water (1:1) mixtures of dry soil and distilled water using a Delta 320 pH meter (Mettler Toledo Instruments (Shanghai, China) Co., Ltd.). Total nitrogen content was measured with Kjeldahl digestion and distillation azotometry. The available phosphorus content was measured by lixiviating-molybdenum blue colorimetry after extraction with 0.5 M NaHCO_3_ (pH 8.5) for 30 min. The available potassium was measured by flame photometer method after extraction with 1 M ammonium acetate.

### 4.4. Data Sources

The crop and soil data were obtained from the National Monitoring Base of Soil Fertility and Fertilizer Efficiency, the Chinese Academy of Agricultural Sciences, and recorded data from the three same long-term experiment sites (1990–2010). Crop data include grain yields and straw yields. The grain and straw yields were the averages of three replicate plots at all sites. The soil data were the chemical analysis values of the soil samples after maize harvesting, including soil organic matter, pH, total nitrogen, available phosphorus, and available potassium.

Climate data were collected from three climate stations of the National Meteorological Centre, Beijing station, Zhengzhou station, and Hengyang station, which were located in or near the experimental sites. Air temperature data (daily mean, maximum and minimum temperatures) and precipitation were obtained from the complete records from 1989 to 2011 of the Chinese Meteorological Administration [49].

### 4.5. Data Analysis 

The mean yields under different fertilization treatments were calculated across the experimental years. Analysis of variance was employed to examine the long-term fertilization effects on wheat and maize yields. We tested the significance of time trends in actual recorded climate data in different treatments at *p* < 0.05 by linear regression analyses. A simple correlation analysis was used to evaluate the relationship between wheat and maize yields and growth-season climate date, thus eliminating the cumulative effects of annual fertilization on both crop yields. Moreover, we used CCA-based variation partitioning analysis (VPA) [50] to reveal the contributions of climate and soil properties changes to the variations in crop yield under long-term fertilization treatments.

#### Variation Partitioning Analysis and Canonical Correspondence Analysis

Variation in crop yield is studied by canonical analysis of the yield data as a function of different types of environmental variables: climate, water, or soil chemistry, environmental impact descriptors, and so on. Variation partitioning is one technique for this type of analysis. CCA is an analysis method based on variation partitioning and is a way to make sense of cross-covariance matrices. CCA identified the subset of variable sets based on Spearman rank correlation.

We divided all the variables into two sets: the climate variables set and the soil variables set. We selected the average temperature, maximum temperature, minimum temperature, and precipitation as the climate variables set. The average temperature affects crop growth and development; the maximum temperature and minimum temperature affect crop photosynthesis, respiration and growth, and development; and precipitation during the growth period affects the utilization of water. For the soil variables set, pH, soil organic matter, soil total nitrogen, available phosphorus, and available potassium were chosen because soil organic matter directly reflects the level of soil fertility, soil total nitrogen provides nitrogen nutrition for the crop, available phosphorus, and available potassium in soil can be directly absorbed by the crop, and pH directly affects the activity of nutrient elements. The selected sets of variables were tested for significance by the CCA model based on 500 permutations using eigenvalues of constrained and unconstrained correspondence axes. Euclidean distances were used to construct similarity matrices based on these identified environmental factors, which were then used to test the effects of these sets of variables on crop yield. CCA was performed in R 2.13.1 using the function ‘‘cca’’ in vegan (Version 1.17–9) and the package CANOCO for verification purposes [51].

## 5. Conclusions

Major Chinese cropping areas have experienced significant climatic warming trends in recent decades. Under the same climatic change, however, crop yields showed dissimilar variation trends due to different fertilization regimes. The differences in crop yield variation trends might be due to soil properties changes and their interactions with climate changes. In fact, the contributions of soil properties changes to crop yield variation were greater than those of climate change, especially under un-sound fertilization practices. Our results highlight that soil properties changes should be considered in the assessment of climate change impacts on crop production. Given the significant contributions of soil properties changes to crop yield variation, the impacts of climate change on crop yield might have been under- or over-estimated in previous assessments. Further field observations are necessary to determine the actual impacts of climate change on crop yield.

## Figures and Tables

**Table 1:** The changes of soil fertility factors under different fertilization regimes in the three experimental sites during 1991–2010.

Station	Treatment	pH		SOM		TN		AP		AK	
		Mean ± SE	Trend	Mean ± SE	Trend	Mean ± SE	Trend	Mean ± SE	Trend	Mean ± SE	Trend
				(g kg^−1^)	(g kg^−1^ a^−1^)	(g kg^−1^)	(g kg^−1^ a^−1^)	(mg kg^−1^)	(mg kg^−1^ a^−1^)	(mg kg^−1^)	(mg kg^−1^ a^−1^)
Changping	CK	8.20 ± 0.04	0.0086	14.94 ± 0.50	0.3024 **	0.66 ± 0.04	−0.0179 **	3.37 ± 0.24	−0.0683	67.28 ± 2.80	−1.402 **
	N	8.09 ± 0.04	−0.007	15.01 ± 0.38	0.2373 **	0.77 ± 0.03	−0.0074	3.05 ± 0.22	−0.1138 **	67.47 ± 2.56	−1.1152 **
	NPK	8.12 ± 0.03	−0.0038	14.56 ± 0.32	0.1344 **	0.79 ± 0.03	0.0037	13.01 ± 1.01	0.2322	77.65 ± 2.45	0.1166
	NPKM	8.03 ± 0.03	−0.0127*	16.38 ± 0.64	0.4083 **	0.88 ± 0.04	0.0091	62.18 ± 11.34	7.5046 **	85.31 ± 2.97	0.8338
Zhengzhou	CK	8.52 ± 0.06	0.0291 **	10.47 ± 0.09	−0.0018	0.67 ± 0.02	0.0122 **	3.33 ± 0.34	−0.1893 **	55.93 ± 1.87	0.0658
	N	8.43 ± 0.04	0.0177 **	10.95 ± 0.15	0.0543 *	0.70 ± 0.02	0.0034	3.42 ± 0.33	−0.1449 **	55.45 ± 1.42	0.0421
	NPK	8.37 ± 0.02	0.0089 *	11.90 ± 0.25	0.1717 **	0.72 ± 0.02	0.0105 **	19.96 ± 1.68	0.8812 **	85.52 ± 5.02	3.2008 **
	NPKM	8.34 ± 0.03	0.0069	14.95 ± 0.56	0.4076 **	0.88 ± 0.04	0.026 **	40.76 ± 5.07	3.5277 **	120.59 ± 10.24	7.1037 **
Qiyang	CK	5.72 ± 0.09	−0.0433 **	15.24 ± 0.26	0.0545	0.79 ± 0.03	−0.0058	4.74 ± 0.46	−0.1466	69.01 ± 7.88	−4.6054 **
	N	4.67 ± 0.19	−0.1194 **	16.27 ± 0.36	0.1819 **	0.98 ± 0.03	0.01	4.25 ± 0.42	−0.0917	58.23 ± 7.95	−3.4518 **
	NPK	4.78 ± 0.13	−0.0736 **	18.19 ± 0.63	0.3902 **	1.06 ± 0.03	0.0031	29.58 ± 3.15	1.4664 **	134.05 ± 7.24	1.4316
	NPKM	5.95 ± 0.07	−0.0214	22.47 ± 1.22	0.7278 **	1.27 ± 0.04	0.0122	101.09 ± 14.12	9.8349 **	224.97 ± 10.77	6.5402 **

SE means standard error, ** significant at *p* < 0.01, * significant at *p* < 0.05.

**Table 2:** Mean yields of wheat and maize and their changing trends affected by long-term fertilization during 1991–2010.

Site	Treatment	Wheat		Maize	
		Mean ± SE	Trend	Mean ± SE	Trend
		(kg ha^−1^)	(kg ha^−1^ a^−1^)	(kg ha^−1^)	(kg ha^−1^ a^−1^)
Changping	CK	537.6 ± 58.3	−34.65 **	1755.8 ± 143.4	−55.94 *
	N	600.5 ± 74.3	−45.56 **	2079.6 ± 242.3	−91.94 *
	NPK	3811.7 ± 225.6	113.78 **	4988.5 ± 265.1	64.24
	NPKM	4250.1 ± 246.9	105.06*	5644.7 ± 283.2	100.01 *
Zhengzhou	CK	1859.0 ± 75.2	−19.96	2817.1 ± 191.5	−83.10 *
	N	2370.9 ± 224.6	−92.89 *	3433.6 ± 274.5	−141.01 **
	NPK	6326.3 ± 160.7	73.20 **	6598.0 ± 367.8	151.60 *
	NPKM	5732.9 ± 174.1	22.23	6826.0 ± 349.3	147.99 *
Qiyang	CK	353.5 ± 28.9	−15.19 **	266.8 ± 47.4	−24.86 **
	N	342.4 ± 93.1	−61.53 **	607.7 ± 183.6	−113.65 **
	NPK	1188.9 ± 110.2	−66.12 **	3048.9 ± 319.4	−161.14 **
	NPKM	1699.6 ± 89.2	8.58	5055.7 ± 241.2	71.99

** Significant at *p* < 0.01; * significant at *p* < 0.05.

**Table 3:** The correlation coefficients between wheat yields and main climatic and soil fertility factors in the three experimental sites.

Site	Treatment	Tavg	Tmax	Tmin	Prec	pH	SOM	TN	AP	AK
Changping	CK	−0.187	−0.109	−0.126	−0.222	−0.490 *	−0.546 *	0.356	0.412	0.413
	N	−0.237	−0.103	−0.228	−0.215	0.11	−0.579 **	0.259	0.585 **	0.287
	NPK	0.131	0.070	0.093	0.127	0.254	0.175	0.354	0.645 **	−0.054
	NPKM	0.224	0.236	0.109	0.045	−0.148	0.495 *	0.403	0.560 *	0.217
Zhengzhou	CK	−0.152	0.112	−0.296	−0.002	0.222	0.324	−0.357	0.011	−0.212
	N	−0.412	−0.100	−0.500 *	0.137	−0.134	−0.614 **	−0.444 *	0.733 **	0.268
	NPK	0.632 **	0.533 *	0.576 **	−0.485 *	0.297	0.452 *	0.243	0.324	0.378
	NPKM	0.277	0.369	0.151	−0.271	−0.049	0.221	0.209	0.234	0.231
Qiyang	CK	−0.523 *	−0.524 *	−0.515 *	0.225	0.421	−0.103	0.289	0.591 **	0.491 *
	N	−0.487 *	−0.510 *	−0.476 *	0.239	0.888 **	−0.631 **	−0.335	0.390	0.672 **
	NPK	−0.472 *	−0.468 *	−0.508 *	0.244	0.768 **	−0.632 **	−0.026	−0.513 *	−0.327
	NPKM	0.001	0.025	−0.014	0.250	0.097	0.077	−0.118	0.076	0.237

Tavg, Tmax, Tmin, and Prec are annual average temperature, maximum temperature, minimum temperatures, and precipitation, respectively. SOM, TN, AP, AK are soil organic matter, total nitrogen, available phosphorus, available potassium, respectively. ** Significant at *p* < 0.01; * significant at *p* < 0.05.

**Table 4:** The correlation coefficients between maize yields and main climatic and soil fertility factors in the three experimental sites.

Site	Treatment	Tavg	Tmax	Tmin	Prec	pH	SOM	TN	AP	AK
Changping	CK	0.018	0.081	−0.128	0.162	−0.064	−0.227	0.549 *	0.359	0.282
	N	−0.023	−0.001	−0.136	0.248	0.071	−0.347	0.248	0.455 *	0.391
	NPK	−0.002	0.13	−0.001	−0.205	0.049	0.194	0.43	0.334	0.173
	NPKM	0.036	0.185	0.005	−0.303	−0.252	0.444 *	0.444 *	0.466 *	0.258
Zhengzhou	CK	−0.418	−0.146	−0.650 **	−0.624 **	−0.410	−0.225	−0.180	0.500 *	−0.093
	N	−0.315	−0.012	−0.552 *	−0.644 **	−0.362	−0.500 *	−0.110	0.769 **	0.220
	NPK	−0.068	−0.167	0.016	0.039	0.108	0.35	0.526 *	0.08	0.482 *
	NPKM	0.041	−0.056	0.104	−0.021	0.058	0.513 *	0.612 **	0.567 **	0.570 **
Qiyang	CK	−0.362	−0.324	−0.547 *	0.165	0.761 **	0.111	−0.024	0.331	0.683 **
	N	−0.236	−0.197	−0.422 *	0.008	0.872 **	−0.509 *	−0.364	0.286	0.663 **
	NPK	−0.355	−0.299	−0.452 *	0.268	0.121	−0.476 *	−0.254	−0.203	−0.104
	NPKM	−0.007	−0.048	0.133	−0.086	−0.541 *	0.524 *	0.248	0.476 *	0.352

Tavg, Tmax, Tmin, and Prec are annual average temperature, maximum temperature, minimum temperatures, and precipitation, respectively. SOM, TN, AP, AK are soil organic matter, total nitrogen, available phosphorus, available potassium, respectively. ** Significant at *p* < 0.01; * significant at *p* < 0.05.

## Data Availability

The data presented in this study are available on request from the corresponding author.

## Supplementary Materials

The following are available online at https://www.mdpi.com/article/10.3390/plants10102002/s1, Figure S1: The spatial locations of the three long-term experimental sites; Figure S2: The monthly mean temperature (A) and precipitation (B) during 1991–2010 of the three long-term experimental sites; Figure S3: Historical trends of annual average, maximum and minimum temperatures in the three experimental sites over the wheat and maize growing seasons during 1991–2010. Straight lines are the linear regression lines. A, wheat of Changping, B, wheat of Zhengzhou, C, wheat of Qiyang, D, maize of Changping, E, maize of Zhengzhou, F, maize of Qiyang. ** Significant at *p* < 0.01; * Significant at *p* < 0.05; Figure S4: Historical trends of precipitation in the three experimental sites over wheat (A) and maize (B) growing seasons during 1991–2010. * Significant at *p* < 0.05; Figure S5: The changes of soil fertility index under different fertilization regimes in the three experimental sites during 1991–2010. SOM, soil organic matter, TN, total nitrogen, AP, available phosphorus, AK, available potassium, ** Significant at *p* < 0.01; * Significant at *p* < 0.05. Table S1: The main climatic index (1980–2010) and the initial soil properties of the three long-term experimental sites (1990); Table S2: Fertilization regimes conducted for wheat and maize cropping in the three long-term experimental sites.
