# Supplementary material for: Contributions of Climate and Soil Properties to Wheat and Maize Yield Based on Long-Term Fertilization Experiments
Source: Plants (Basel). 2021 Sep 24;10(10):2002. doi: 10.3390/plants10102002 (PMC8537906; doi:10.3390/plants10102002)
Supplement: Supplementary file 1 [file plants-10-02002-s001.zip › plants-1369534-supplementary.pdf]

Supporting information for

Contributions of climate and soil fertility to wheat and maize yield based on long-term fertilization experiments

Shengbao Wei, Anchun, Peng Xiaomin Huang, Aixing Deng, Changqing Chen, Weijian Zhang

Supplemental figures

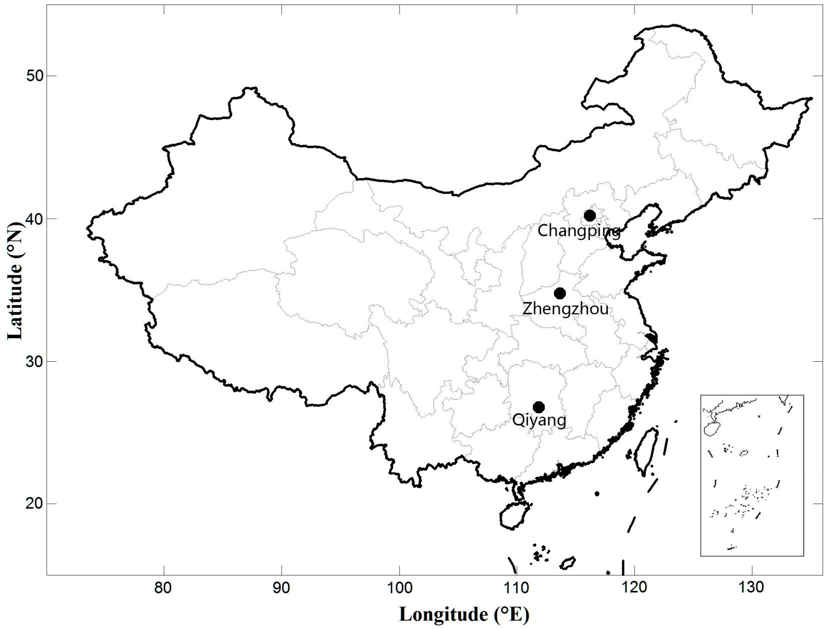

Fig. S1 The spatial locations of the three long-term experimental sites.

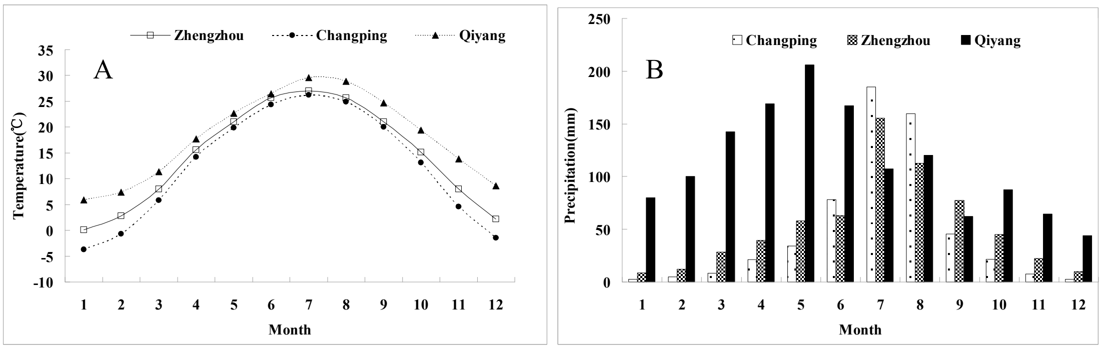

Fig. S2 The monthly mean temperature (A) and precipitation (B) during 1991-2010 of the three long-term experimental sites.

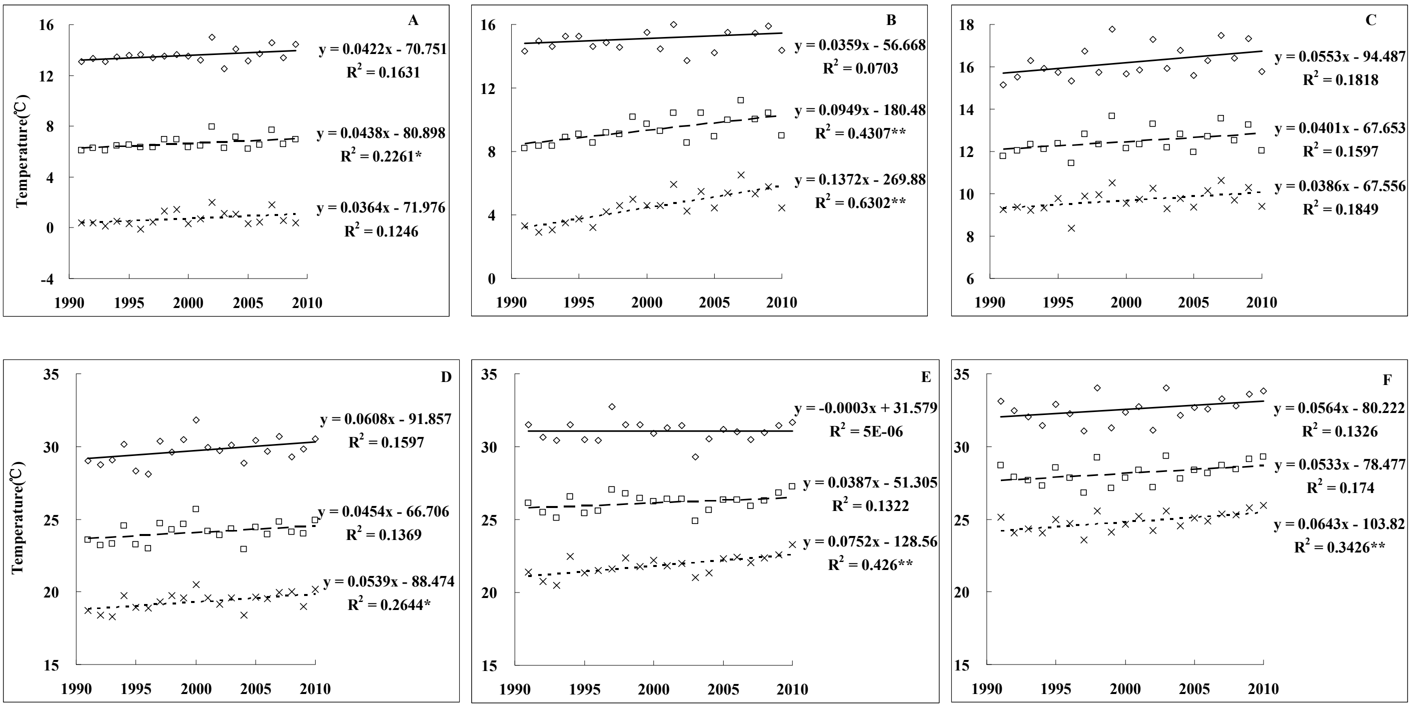

Fig. S3 Historical trends of annual average, maximum and minimum temperatures in the three experimental sites over the wheat and maize growing seasons during 1991-2010. Straight lines are the linear regression lines. A, wheat of Changping, B, wheat of Zhengzhou, C, wheat of Qiyang, D, maize of Changping, E, maize of Zhengzhou, F, maize of Qiyang. ** Significant at P<0.01; * Significant at P<0.05

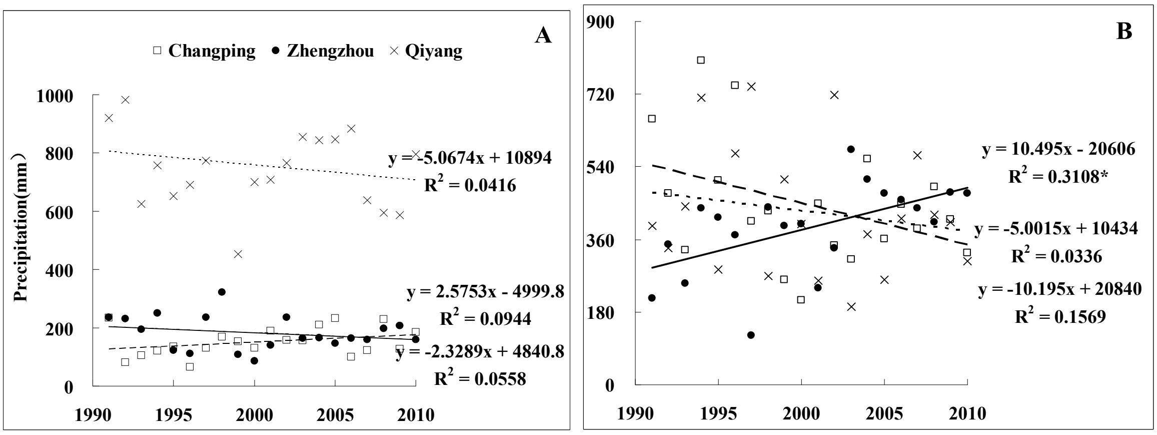

Fig. S4 Historical trends of precipitation in the three experimental sites over wheat (A) and maize (B) growing seasons during 1991-2010. * Significant at P<0.05.

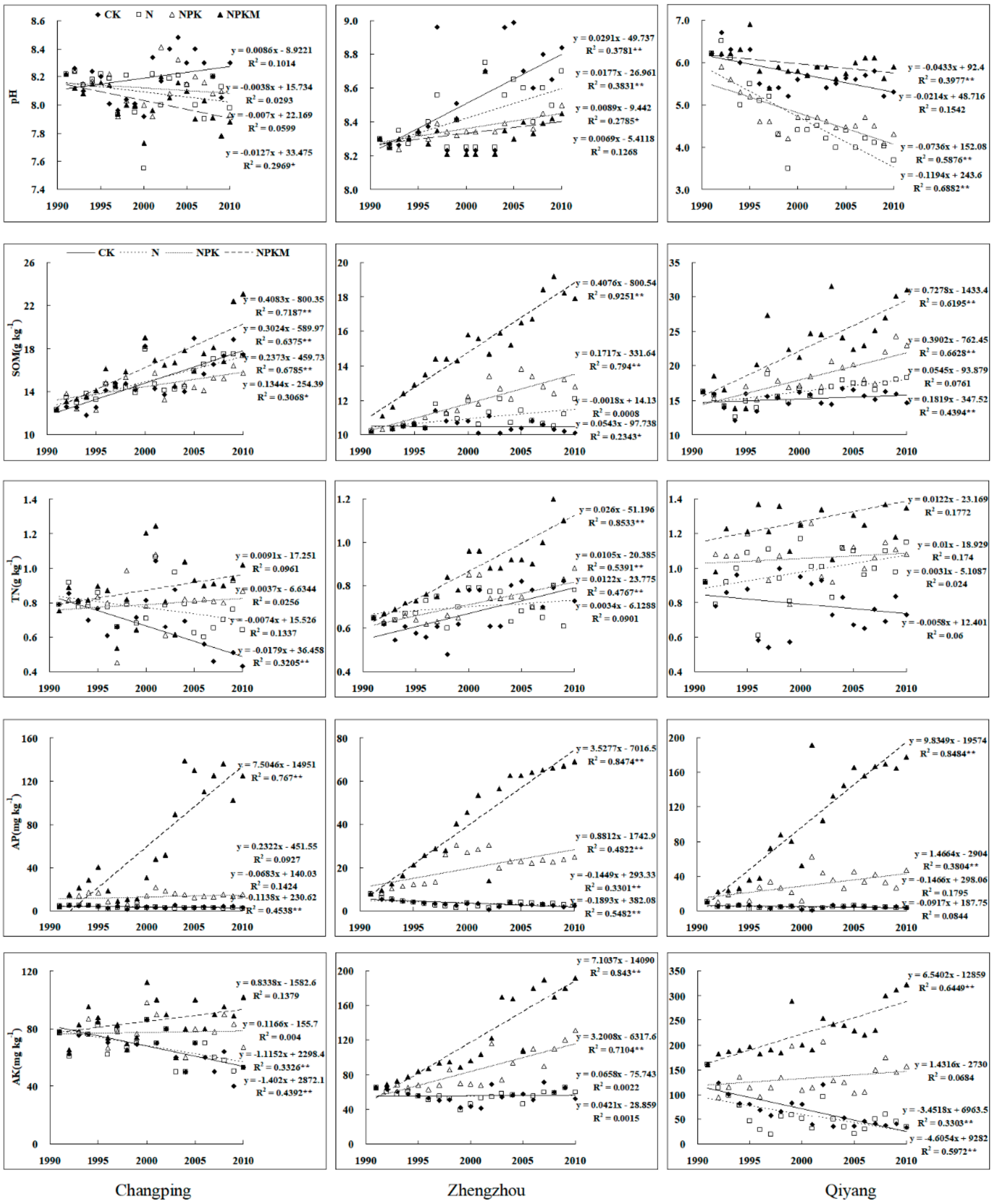

Fig. S5 The changes of soil fertility index under different fertilization regimes in the three experimental sites during 1991-2010. SOM, soil organic matter, TN, total nitrogen, AP, available phosphorus, AK, available potassium, ** Significant at P<0.01; * Significant at P<0.05.

Supplemental Tables

Table S1 The main climatic index (1980-2010) and the initial soil properties of the three long-term experimental sites (1990).

Item	Changping	Zhengzhou	Qiyang
Spatial location	E115°50′~116°29′ N 40°2′~40°23′	E112°42'~114°13' N34°16'~34°58'	E111°35'~112°14' N26°02'~26°51'
Annual mean temperature(℃)	11.8	14.4	18.2
Annual mean precipitation (mm)	550.3	640.9	1,200.0
Annual days of frostless (d)	163	220	291
Soil type	Aquic cinnamon soil	Fluvo-aquic soil	Red Soil
Soil organic matter (g kg^{-1})	11.5	10.2	12.3
Soil total N (g kg^{-1})	1.07	0.65	0.80
Soil available P (mg kg^{-1})	13.9	7.7	11.2
Soil available K (mg kg^{-1})	104.0	65.0	78.6
Soil pH	8.8	8.3	5.7

Table S2 Fertilization regimes conducted for wheat and maize cropping in the three long-term experimental sites.

Experimental site	Crop	Nutrient	Treatments (kg ha^{-1})				
			CK	N	NPK	NPKM(fertilizers+ manure) Nutrients from fertilizers	NPKM(fertilizers+ manure) Nutrients from manure
Changping	Wheat	N	0	150	150	150	70.1
		P_2O_5	0	0	75	75	106.6
		K_2O	0	0	45	45	116.3
	Maize	N	0	150	150	150	70.1
		P_2O_5	0	0	75	75	106.6
		K_2O	0	0	45	45	116.3
Zhengzhou	Wheat	N	0	165	165	49.5	115.5
		P_2O_5	0	0	82.5	44.0	38.5
		K_2O	0	0	82.5	18.3	64.2
	Maize	N	0	187.5	187.5	56.5	131
		P_2O_5	0	0	93.5	49.7	43.8
		K_2O	0	0	93.5	20.7	72.8
Qiyang	Wheat	N	0	90	90	27	63
		P_2O_5	0	0	36	36	32
		K_2O	0	0	36	36	30
	Maize	N	0	210	210	63	147
		P_2O_5	0	0	84	84	75
		K_2O	0	0	84	84	70

*The long-term experiment sites receive 6.7-13.2 t ha^{-1} of swine manure in Qiyang site, and 30-75 t ha^{-1} of compost in Zhengzhou and Changping sites
